# FlexDotPlot: a universal and modular dot plot visualization tool for complex multifaceted data

**DOI:** 10.1093/bioadv/vbac019

**Published:** 2022-03-23

**Authors:** Simon Leonard, Aurélie Lardenois, Karin Tarte, Antoine D Rolland, Frédéric Chalmel

**Affiliations:** 1 Univ Rennes, Inserm, EHESP, Irset (Institut de recherche en santé, environnement et travail), UMR_S 1085, F-35000 Rennes, France; 2 UMR 1236, University of Rennes, INSERM, Etablissement Français du Sang Bretagne, F-35043 Rennes, France; 3 LabEx IGO “Immunotherapy, Graft, Oncology”, F-35043 Nantes, France; 4 SITI Laboratory, Etablissement Français du Sang Bretagne, CHU Rennes, Rennes, F-35033 France

## Abstract

**Motivation:**

Dot plots are heatmap-like charts that provide a compact way to simultaneously display two quantitative information by means of dots of different sizes and colors. Despite the popularity of this visualization method, particularly in single-cell RNA-sequencing (scRNA-seq) studies, existing tools used to make dot plots are limited in terms of functionality and usability.

**Results:**

We developed FlexDotPlot, an R package for generating dot plots from multifaceted data, including scRNA-seq data. It provides a universal and easy-to-use solution with a high versatility. An interactive R Shiny application is also available allowing non-R users to easily generate dot plots with several tunable parameters.

**Availability and implementation:**

Source code and detailed manual are available on CRAN (stable version) and at https://github.com/Simon-Leonard/FlexDotPlot (development version). Code to reproduce figures is available at https://github.com/Simon-Leonard/FlexDotPlot_paper. A Shiny app is available as a stand-alone application within the package.

**Supplementary information:**

Supplementary data are available at *Bioinformatics Advances* online.

## 1 Introduction

Data visualization is essential for the biological mining of the vast amount of information generated by high-throughput technologies. One of the most popular plotting techniques in genomics is the heatmap. It is used to display a single quantitative information from a data matrix (Wilkinson and Friendly, 2009). For instance, in transcriptomics, rows and columns usually represent genes and samples, respectively, and boxes are color-coded according to expression signals. However, heatmaps appear intrinsically limited to provide comprehensive representation of the diversity of data now available from single-cell technologies. Accordingly, alternative methods such as dot (or spot) plots are increasingly used.

A dot plot is a modified heatmap where each box in the grid is replaced by a dot. In addition to quantitative values that are displayed via a color gradient, the dot size is also used to represent another quantitative information, e.g. the fraction of cells expressing a given gene within a single-cell RNA-sequencing (scRNA-seq) dataset (Habib *et al.*, 2017; Lukassen *et al.*, 2018; Ordovas-Montanes *et al.*, 2018; Wu *et al.*, 2018). Although simple dot plots can be easily generated with basic graphics commands (with ggplot2 for example), it becomes more complex and time-consuming when more elaborated plots are needed (with multiple and customized layers/legends such as dendrograms).

In recent years, multiple tools have been developed to make dot plots like the *rain plot* method (Henglin *et al.*, 2019) or the *corrplot* function of the feature-expression heatmap method (Benno Haarman *et al.*, 2015; Wei and Simko, 2021). Several programs dedicated to scRNA-seq analysis (Seurat, scClustViz or cellphonedb) also provide a dot plot function (Efremova *et al.*, 2020; Innes and Bader, 2019; Stuart *et al.*, 2019). A dot plot generator is also available in ProHits-viz, a web-tool dedicated to protein–protein interaction analysis (Knight *et al.*, 2017).

While the increasing number of dedicated tools embedding dot plot representation clearly illustrates the relevance of these visualization methods in current data analysis, elaborated dot plots remain difficult to generate partly due to the lack of universality. In particular, most of the previously mentioned packages use very different input data formats: *rain plot* and *corrplot* are restricted to correlation and association matrixes respectively; cellphonedb, Seurat and scClustViz require an output from their own pipeline with a specific format; ProHits-viz requires quantitative information on bait-prey interaction. In addition, all of the existing methods can only display two quantitative information per dot (Supplementary Table S1). To address these issues, we developed a new tool for dot plot visualization. The main interests of this tool rely on its universality (input can represent different types of data and variables, with a standard data frame format), its customizability (users can specify which variable to display and how each variable should be displayed) and a new way to represent quantitative or qualitative variables in dot plots by using variable shapes.

## 2 Implementation

**Fig. 1.**
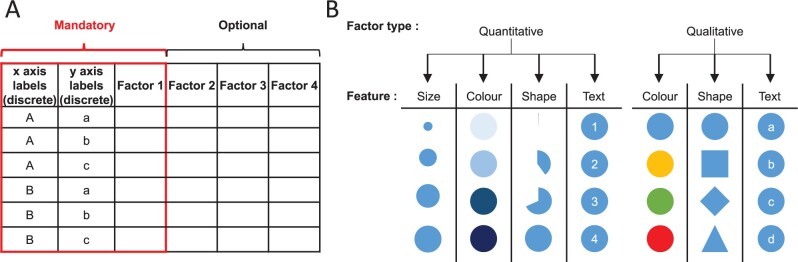
Input format and feature representation. (**A**) The input format corresponds to a table containing a combination of two qualitative variables (used as *x* and *y* axis labels) and at least one factor describing these combinations. Up to three additional factors can be optionally provided and used for the dot plot. (**B**) Description of how a factor is represented in a dot plot according to its type (qualitive or quantitative) and assigned dot feature. Size feature requires a quantitative factor while other features are compatible with any type with adapted rendering

FlexDotPlot is implemented in R and takes advantage of several publicly available R packages for data visualization, manipulation and analysis (Supplementary Table S2). It requires data in standardized input format (data frame) as input: the first two columns contain the two factors to spread along the *x* and *y* axes (e.g. genes and cell populations for scRNA-seq datasets), followed by the corresponding quantitative and/or qualitative data to be displayed (Fig. 1A). From this input, dot plots can be produced with a single command line or interactively with a Shiny application.

## 3 Features highlights

**Fig. 2.**
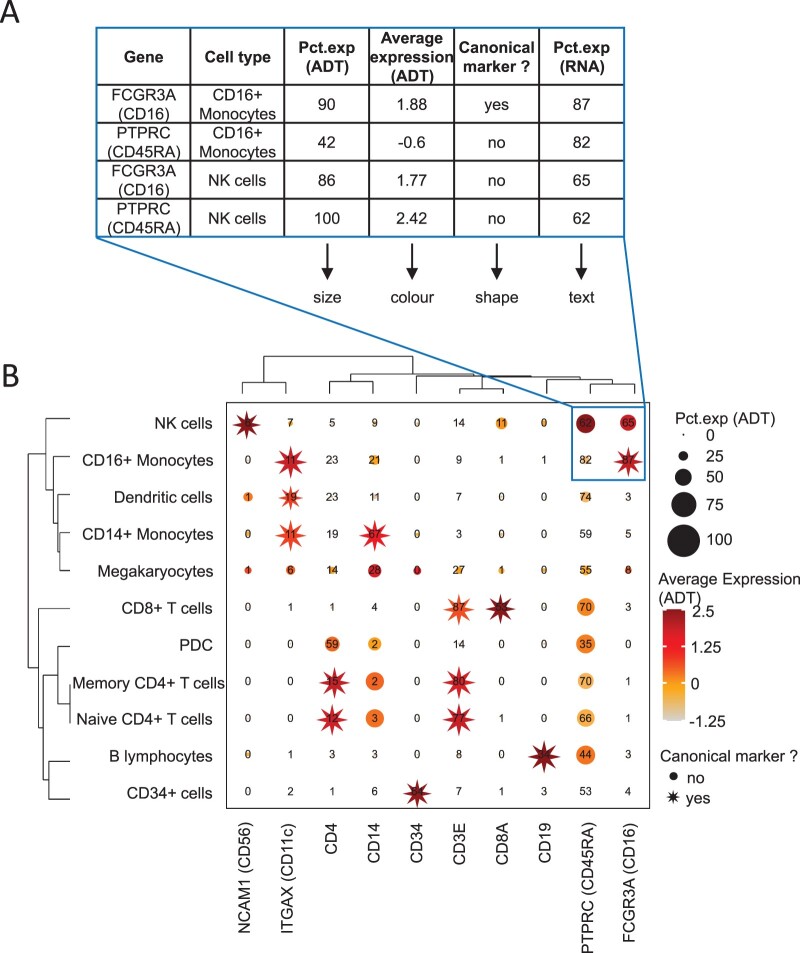
Dot plot representation applied to the 8k human CBMC scRNA-seq dataset. (**A**) Part of the input table used for generating dot plot in B. (**B**) Dot plot on the CBMC dataset with FlexDotPlot. Each dot represents multiple features for one gene (vertical axis) in a given cell cluster (horizontal axis). The size and text of each dot represent the percentage of cells exprsseing a given marker (Pct.exp) according to Antibody Derived Tags (ADT) and RNA level respectively. The color represents the average scaled expression of a given marker at the ADT level. The shape of the dot is used to highlight canonical marker of specific cell types. The top right border highlights the part of the plot corresponding to the table in A

FlexDotPlot consists of a single function to generate dot plots with several easy-to-tune parameters allowing users to specify which and how information has to be represented (Fig. 1B). In addition to the traditional size and color features, users can display two additional information by adding some text or by using dot shapes (Fig. 2). To fit with the customizability of the tool, only one variable controlling one feature is needed and all the other parameters are optional.

One major improvement of FlexDotPlot relies on the possibility to represent a defined factor by using variable dot shapes, which is not possible with other existing dot plot representation methods (Supplementary Table S1). This characteristic is really suitable to represent percentages as it is classically done in scRNA-seq-related dot plots (Supplementary Fig. S1).

Several minor parameters are implemented in the function to easily custom the plot legends, shapes, colors and/or labels. Moreover, a *ggplot2* object can be returned so that users can customize even further the output by adding custom layers if needed.

FlexDotPlot also provides parameters to enhance the resulting figure with dendrograms, which is rarely available in existing methods (Supplementary Table S1) (Wilk *et al.*, 2020). Depending on the variable types, a principal component analysis (quantitative variables), a multiple correspondence analysis (qualitative variables) or a factor analysis for mixed data (quantitative and qualitative variables) is performed prior to clustering analysis.

To further illustrate the versatility and usefulness of FlexDotPlot in representing multimodal data, we used FlexDotPlot on several scRNA-seq datasets combined with complementary results including CITE-seq, copy number variation (CNV) or cell communication data (Bi *et al.*, 2021; Stoeckius *et al.*, 2017; Tirosh *et al.*, 2016). Particularly, we reproduced dot plot figures from published articles or tutorials and proposed enhanced versions using FlexDotPlot (Supplementary Figs. S1–S3).

The first example (PBMC dataset) illustrates how dot plots are commonly used with scRNA-seq datasets (Supplementary Fig. S1A). Instead of using dot size to represent the percentage of cells expressing a given gene, one can illustrate this parameter by using the shape feature (Supplementary Fig. S1B). Row and column dendrograms are also added to this plot.

The second example is also based on a classical dot plot representation (Supplementary Fig. S2). Here, we keep the common features (size to represent the percentage of cells expressing a gene and color to represent the average scaled expression) and take advantage of the shape feature to represent the CNV status of each gene in each patient.

The third example proposes a more detailed way to represent cell communication data (Supplementary Fig. S3). Compared with a dot plot from CellPhoneDB, we propose to split each column in two to illustrate the expression level of each gene in each cell population, revealing some disparities that could not be seen in the classical representation. As shown in the CellPhoneDB dot plot (Supplementary Fig. S3A), the *CD74/MIF* interaction is equally highlighted in three couples of cell populations (TAM/TP1, TAM/TP2 and TAM/CD8+ T cells). However, the expression of *MIF* is far more pronounced in TP2 cells than in TP1 and CD8+ T cells (Supplementary Fig. S3B) suggesting that this interaction may be more relevant for the TAM/TP2 couple than for the two others (TAM/TP1 and TAM/CD8+ T cells).

## 4 Conclusion

FlexDotPlot is an easy-to-use tool for generating highly customizable dot plot representations. It uses standardized input and output formats (data frame and *ggplot2* object, respectively), which also makes the combination of FlexDotPlot and other R pipelines possible. A set of vignettes is provided with the package to illustrate the dot plot construction procedures and their associated parameters.

## Supplementary Material

vbac019_Supplementary_Data
