# Supplementary material for: FlexDotPlot: a universal and modular dot plot visualization tool for complex multifaceted data
Source: Bioinform Adv. 2022 Mar 23;2(1):vbac019. doi: 10.1093/bioadv/vbac019 (PMC9710660; doi:10.1093/bioadv/vbac019)
Supplement: vbac019_Supplementary_Data [file vbac019_supplementary_data.zip › FDP_figure_supp_v6.pdf]

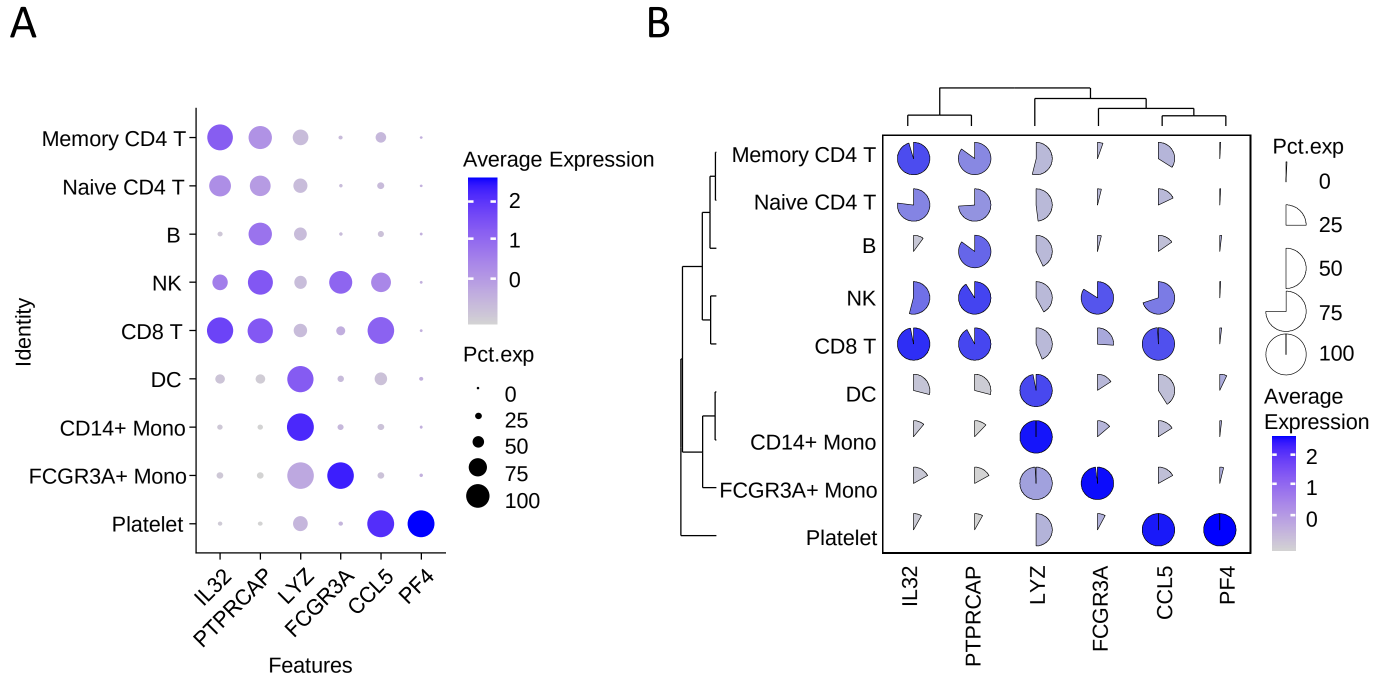

Supplementary Figure 1 : DotPlot representation applied to the 3k human PBMC scRNA-seq dataset.

A. Classical dotplot representation generated with Seurat package. The size of each dot represents the percentage of cells expressing a gene in each cluster (Pct.exp) and the colour represents the average scaled expression of this gene.

B. Improved dotplot representation with FlexDotPlot**.** The percentage of cells expressing a gene is assigned to dot shapes instead of sizes allowing a visual estimation of each value (full circle corresponds to 100%, three quarter circle to 75%, half circle to 50%, quarter circle to 25%) (Kosara, 2019).

Data source : SeuratData package (https://github.com/satijalab/seurat-data)

Script : https://github.com/Simon-Leonard/FlexDotPlot_paper/

Reference : Kosara,R. (2019) Circular Part-to-Whole Charts Using the Area Visual Cue. EuroVis 2019 - Short Papers, 13-17.

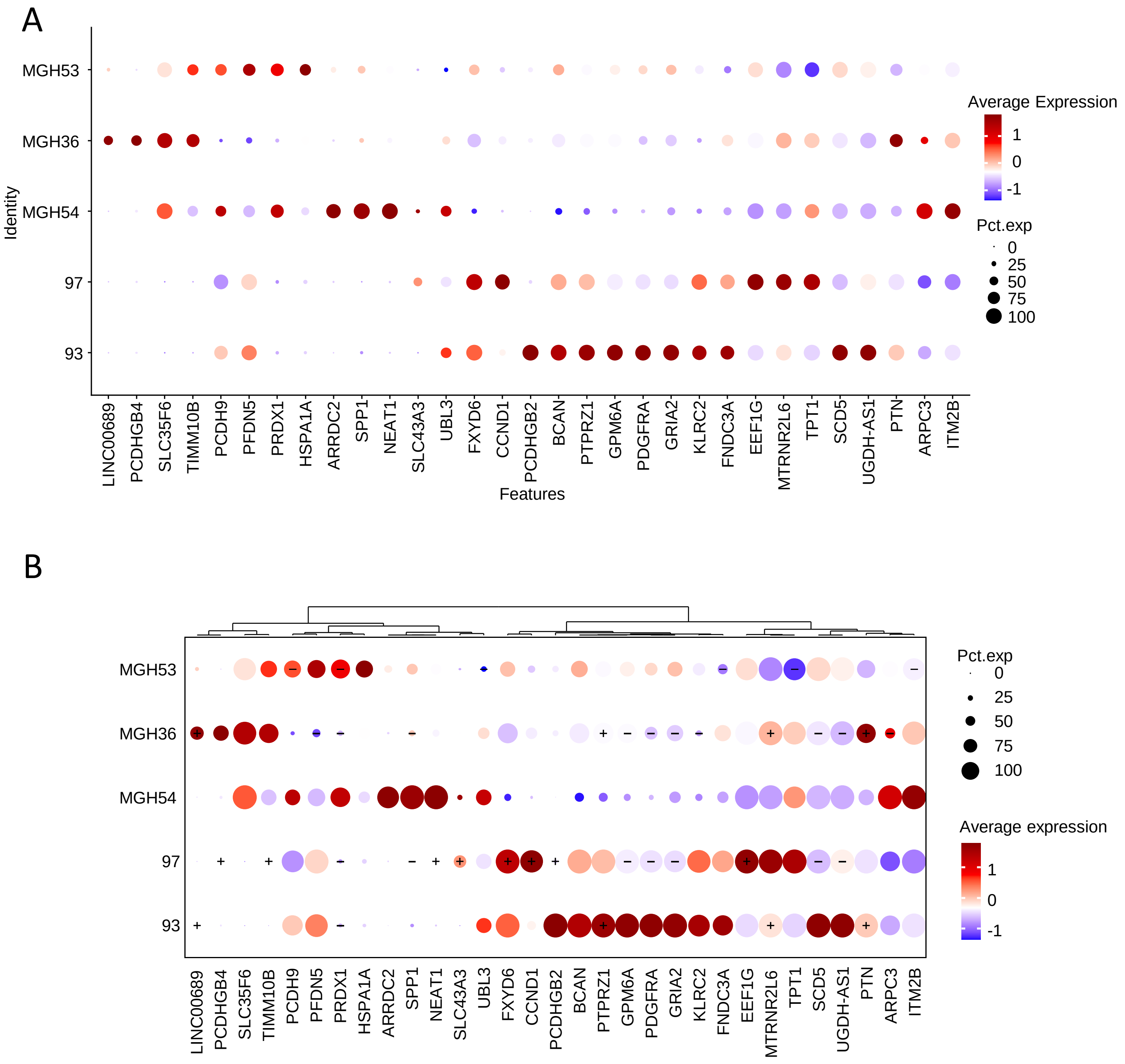

Supplementary Figure 2 : DotPlot representation on InferCNV results from the oligodendroglioma scRNA-seq dataset published by Tirosh et al., 2016.

A. Classical dotplot representation generated with Seurat package. The size of each dot represents the percentage of cells expressing a gene in each cluster (Pct.exp) and the colour represents the average scaled expression of this gene.

B. Improved dotplot representation with FlexDotPlot showing relation between gene expression (dot size and colour) and cnv status determined with InferCNV (text on shape; “+” means up and “-” means down).

Data source : InferCNV package (https://github.com/broadinstitute/inferCNV/)

Script : https://github.com/Simon-Leonard/FlexDotPlot_paper/

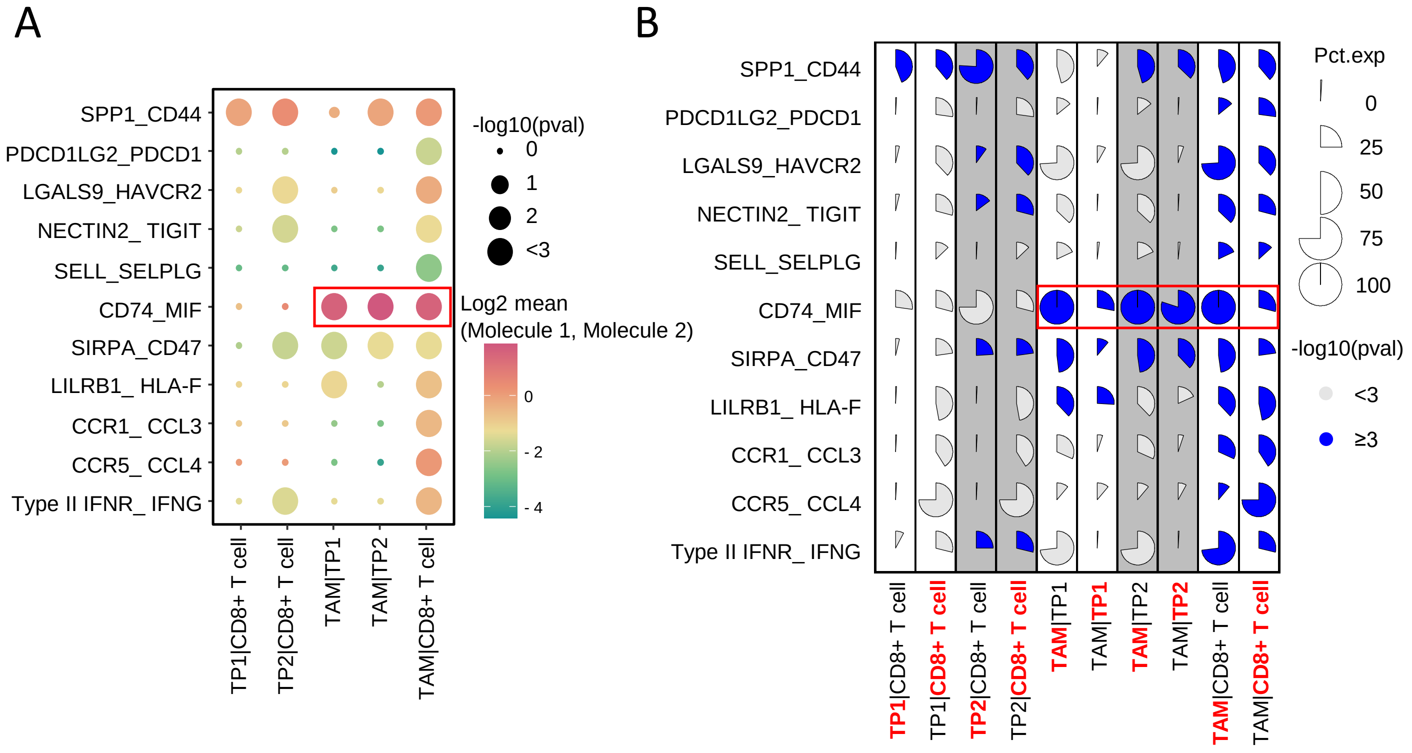

Supplementary Figure 3 : DotPlot representation on CellPhoneDB results from the renal cell carcinoma scRNA-seq dataset published by Bi et al., 2021.

A. Classical dotplot representation generated with CellPhoneDB. The size of each dot represents the significance of interaction and colour represents mean expression of receptor and ligand genes for each pair.

B. Improved dotplot representation with FlexDotPlot**.** Each column from A is splitted into two (labeled with white and grey areas) to show expression metric (percentage of cells expressing a gene) of each ligand/receptor in each cell type. Colour represents the significance of interaction. X axis labels were manually colored in red after plot export. The FlexDotPlot representation unveils some disparities in term of ligand/receptor expression intensities as compared to the classical dotplot (red rectangle in the panel A).

Data source : Bi,K. et al. (2021) Tumor and immune reprogramming during immunotherapy in advanced renal cell carcinoma. Cancer Cell, 39, 649-661.e5.

Script : https://github.com/Simon-Leonard/FlexDotPlot_paper/
